# Neovascular Glaucoma With Persistent Epithelial Defect: Pathophysiology and Surgical Solutions

**DOI:** 10.7759/cureus.84275

**Published:** 2025-05-17

**Authors:** Anugya Sharma, Suneeta Dubey

**Affiliations:** 1 Department of Glaucoma, Dr. Shroff's Charity Eye Hospital, New Delhi, IND

**Keywords:** amniotic membrane grafting, drug-induced toxicity, glaucoma, neovascular glaucoma, persistent epithelial defect, trabeculectomy

## Abstract

We report a unique case of a 55-year-old male diabetic patient who presented with uncontrolled neovascular glaucoma and drug-related surface toxicity, causing persistent epithelial defect in both eyes, and the distinctive management that followed. The successful management of the first eye with amniotic membrane grafting following trabeculectomy prompted a similar approach in the other eye with co-management of both conditions. This case highlights the importance of a tailored, comprehensive approach to such complicated cases.

## Introduction

Neovascular glaucoma (NVG) presents as a refractory glaucoma associated with high failure rates following surgery [1,2]. The refractory nature of the disease and the high failure rate following surgery make the management of the disease more challenging.

Topical glaucoma medications are the standard initial treatment of glaucoma, and 50-60% of the patients on topical antihypertensives are known to have an ocular surface disease [3-6]. The topical glaucoma medications are known to cause burning, stinging, and congestion along with decreased basal tear production and corneal epithelial fluorescein staining [3,7].

We present a unique case of NVG presenting with a persistent epithelial defect and its distinctive management that followed.

## Case presentation

A 55-year-old male patient who was a known diabetic with uncontrolled blood sugars for 12 years presented in the outpatient department. The patient had a history of one intravitreal bevacizumab injection being given in both eyes two months back, followed by a complete pan-retinal photocoagulation (PRP) (two sittings) done elsewhere. At presentation, he had a visual acuity of 6/6 in the right eye (RE) and 6/12 in the left eye (LE) with an intraocular pressure (IOP) of 10 mmHg in RE and 28 mmHg in LE on five topical glaucoma medications with oral acetazolamide. On evaluation, the RE anterior segment evaluation was unremarkable, with the LE showing a central epithelial defect (1 mm x 1 mm), with fine neovascularization of the iris (NVI) at the pupillary margin. Both eyes were pseudophakic with 360-degree synechial angle closure on gonioscopy and a cup disc ratio (CDR) of 0.6:1 in both eyes. Old records revealed that the epithelial defect had been persistent for three months. The patient was initially given frequent lubricants, along with bandage contact lens (BCL) application and shifted to preservative-free medications for a month; however, the epithelial defect showed no signs of healing, and the IOP remained uncontrolled.

**Figure 1:**
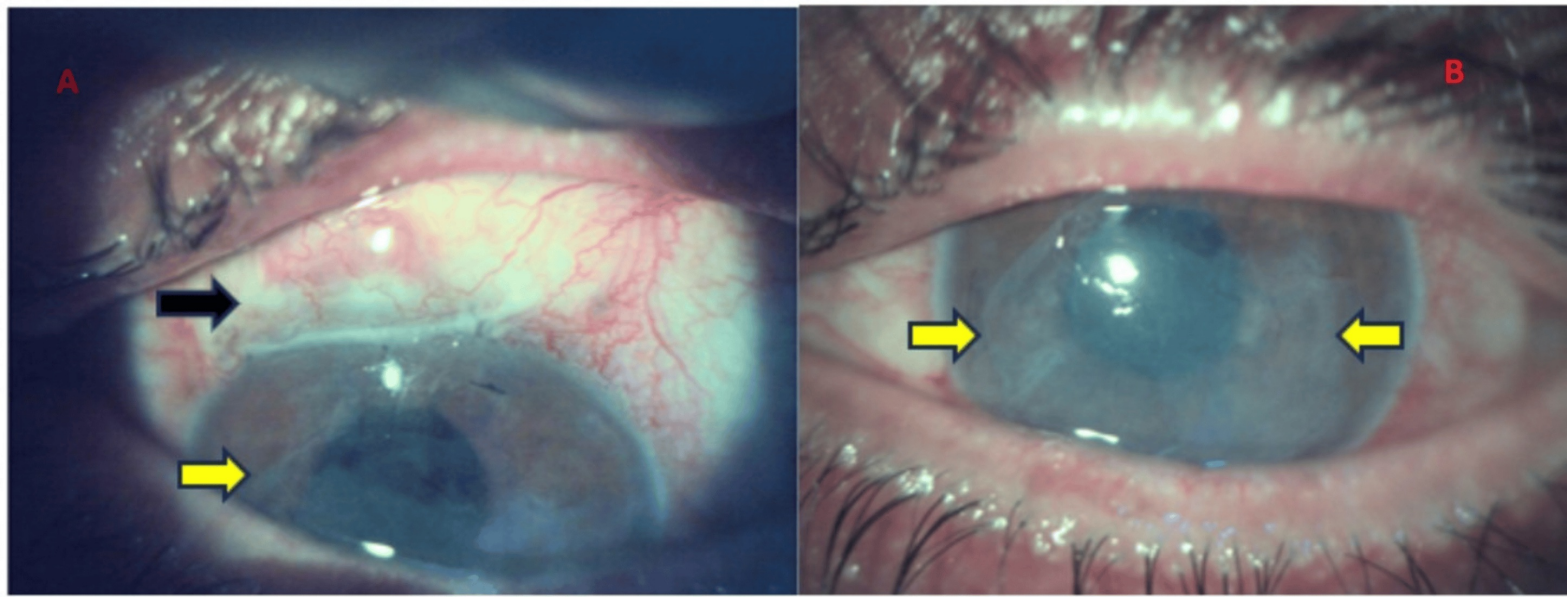
(A, B) Left eye post-amniotic membrane graft application (postoperative week one). Yellow arrows - dissolving the amniotic membrane graft, Black arrow - trabeculectomy bleb. Postoperative week one following amniotic membrane grafting in the left eye. The figure shows a partially dissolved amniotic membrane graft (yellow arrows) and a functioning trabeculectomy bleb (black arrow). The amniotic membrane completely dissolved in the following three weeks, with a healed epithelial defect.

In view of the uncontrolled IOP on maximum therapy with a persistent epithelial defect and NVI, the patient underwent LE trabeculectomy with mitomycin C (MMC) (0.2 mg/mL for two minutes) with tarsorrhaphy and intracameral bevacizumab (1.25 mg/0.05 mL). The surgery was uneventful; however, following surgery, the epithelial defect remained persistent and further enlarged in size, prompting us to do an amniotic membrane grafting (AMG) with BCL application within one week of the primary surgery. Following this, the patient showed dramatic improvement with the resolution of the epithelial defect and good control of IOP (range: 10-14 mmHg) (Figures 1A-1B).

**Figure 2:**
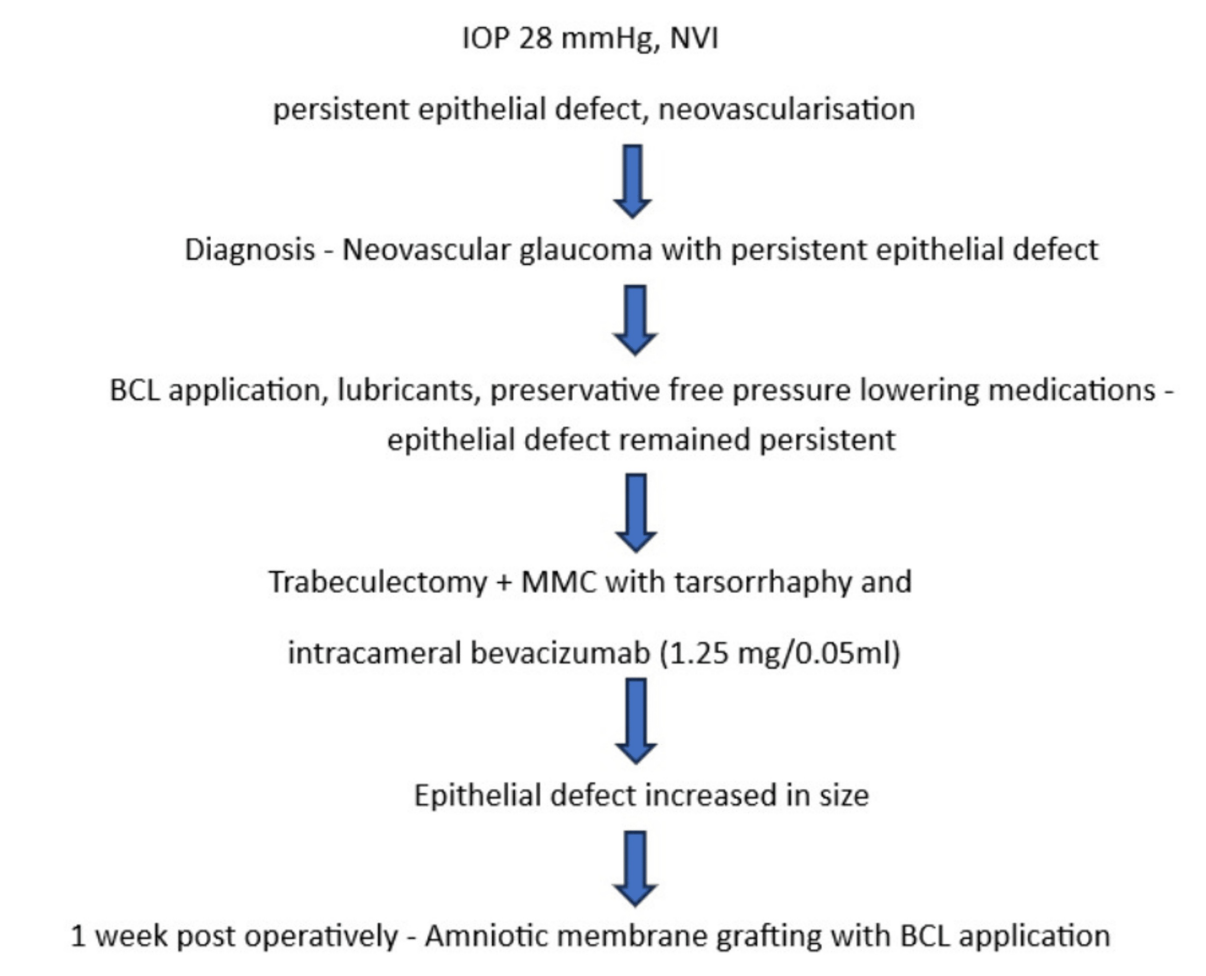
Management of the left eye. BCL: bandage contact lens; MMC: mitomycin C; NVI: neovascularization of the iris; IOP: intraocular pressure

The management of the LE is summarized in Figure 2. 

**Figure 3:**
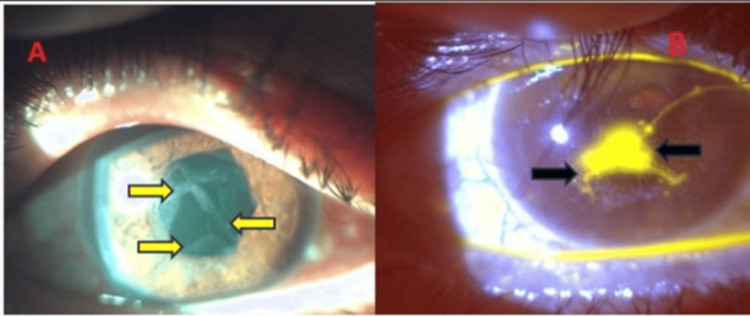
Epithelial defect in the right eye. (A) epithelial defect - approximately 2 x 3 mm in size, with the surrounding loose epithelium (yellow arrows). (B) Fluorescein staining (black arrows) of the epithelial defect. Right eye developed an epithelial defect measuring 2 x 3 mm with the surrounding loose epithelium, as seen in Figure 3A. Figure 3B represents an fluorescein-stained image of the same epithelial defect. This defect did not decrease in size after three months of using lubricants and BCL and remained persistent.

Seven months later, the patient presented with similar complaints of pain, redness, and diminution of vision in the RE with a visual acuity of 2/60 and an uncontrolled IOP (26 mmHg) on maximum medical therapy with fine NVI, a 1 mm hyphaema, and a central epithelial defect (2 x 3 mm) with surrounding loose epithelium (Figures 3A-3B).

Initial management included the application of a BCL and substitution of all topical medications with preservative-free formulations, with the objective of promoting re-epithelialization of the corneal defect. However, this epithelial defect remained persistent despite a BCL application for three months. IOP in the RE also remained uncontrolled on five medications, including oral acetazolamide, while the cupping progressed to 0.9:1 in the RE.

**Figure 4:**
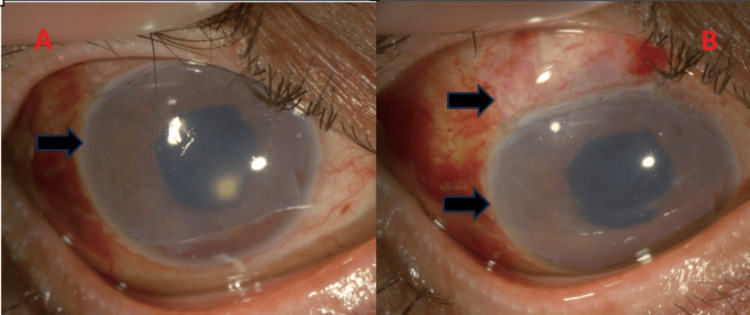
Right post trabeculectomy with amniotic membrane grafting postoperative one week. A) Black arrow - transparent amniotic membrane over the cornea. B) Black arrows - bleb and cornea covered by amniotic membrane

**Figure 5:**
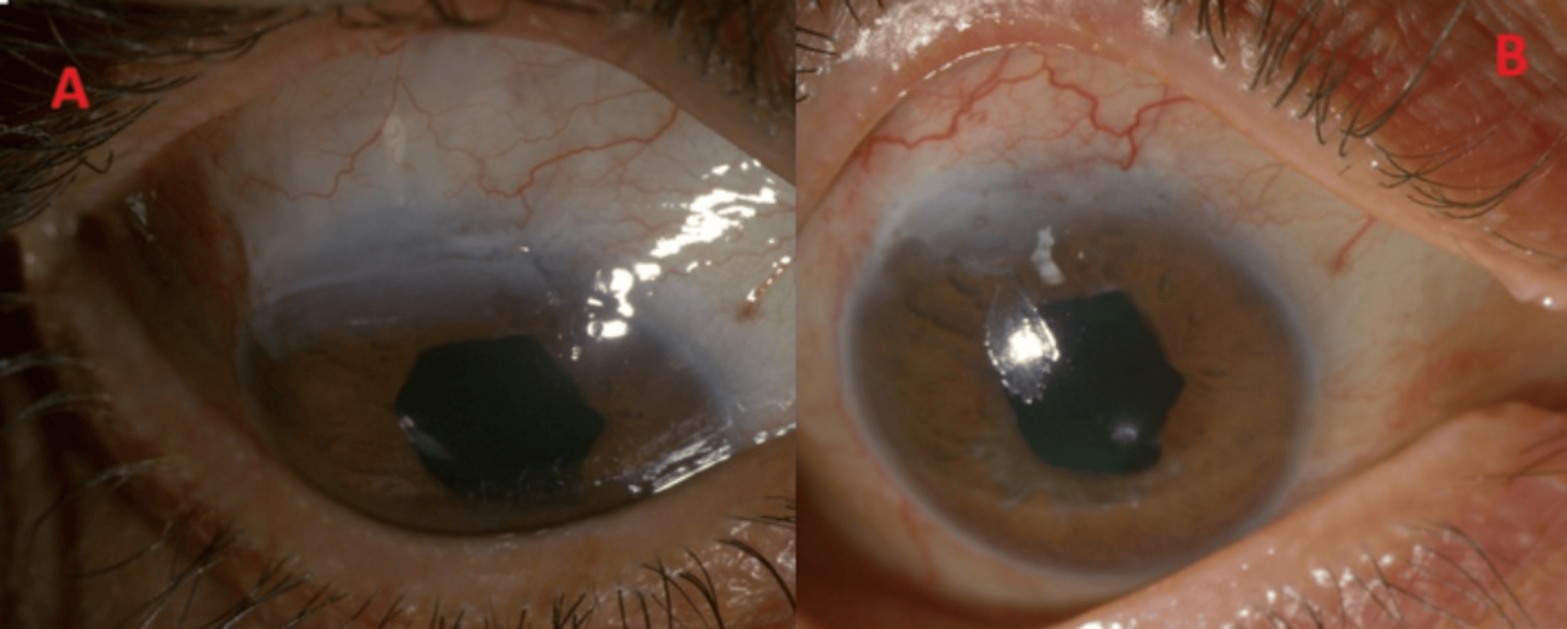
(A) Three weeks post surgery - clear cornea with amniotic membrane graft dissolved. (B) At five months post surgery - healthy ocular surface.

Therefore, taking cues from his other eye, it was decided that trabeculectomy with MMC and AMG should be done. Following an uneventful trabeculectomy with MMC, corneal debridement was done, and AMG was applied using the fibrin glue, followed by a large-diameter BCL application. Postoperatively, the AMG started dissolving in one week (Figure 4) and completely dissolved in three weeks with a completely healed epithelial defect (Figure 5).

**Figure 6:**
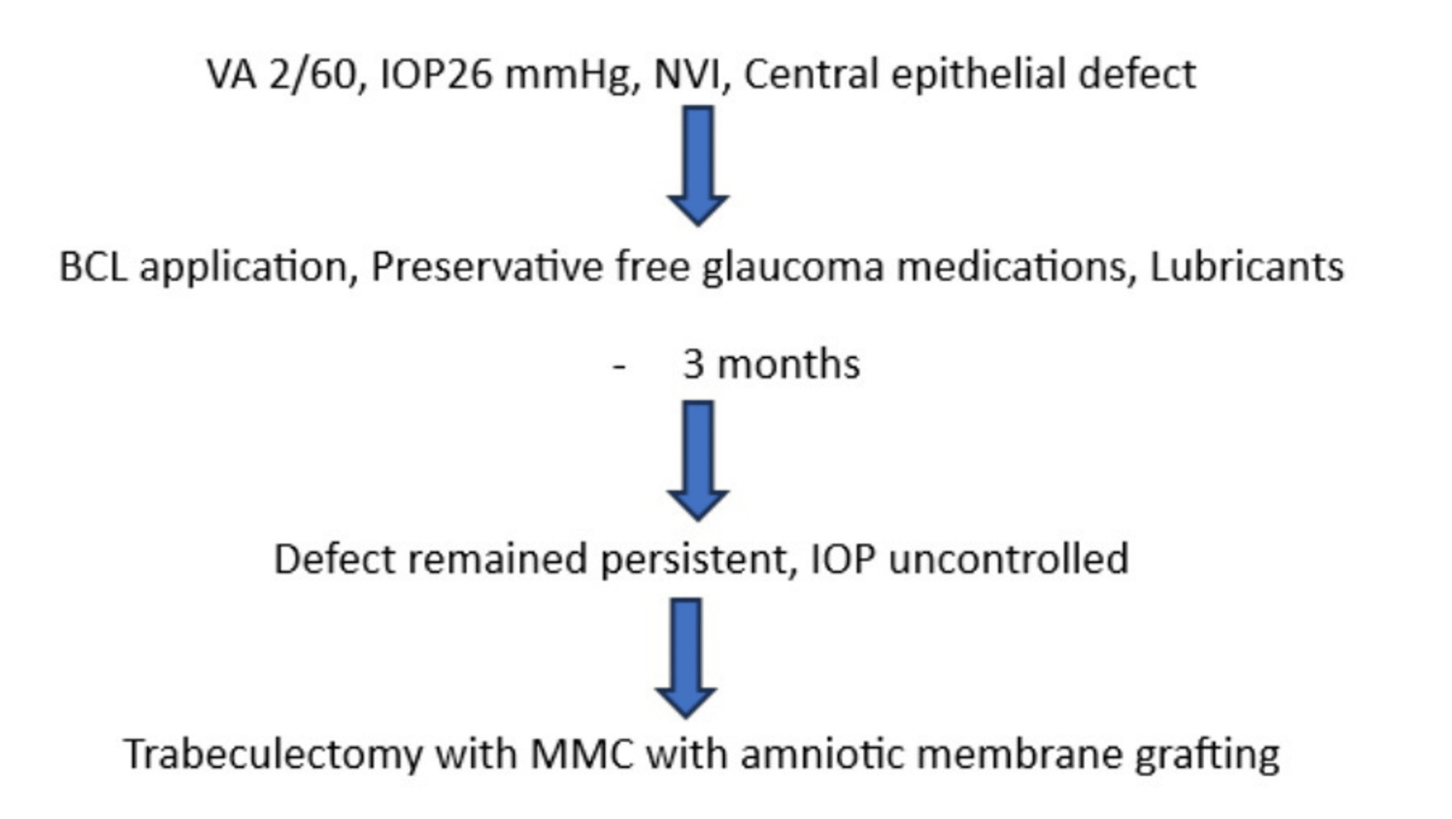
Management of the right eye. BCL: bandage contact lens: MMC: mitomycin C; NVI: neovascularization of the iris; IOP: intraocular pressure

Figure 6 summarizes the management pathway for RE. 

At six months after surgery, the patient is doing well, with an IOP of 10 mmHg in RE and 12 mmHg in LE (11 months after surgery) on one antiglaucoma medications in the LE, with a VA of 6/24 in RE and 6/9 in LE.

## Discussion

Neovascular glaucoma is known to be a potentially blinding secondary glaucoma, characteristically presenting with features such as neovascularization of the iris, elevated IOP, redness, and pain [8]. In this case, neovascular glaucoma presenting with uncontrolled IOP and persistent epithelial defect posed a unique management challenge. An extensive literature search did not reveal such similarly co-managed cases.

The high risk of failure makes the surgical management of NVG challenging. Success rates following trabeculectomy vary between 60% and 80% at one year and 50% at five years across different studies [8,9]. A good prior PRP with anti-vascular endothelial growth factor (VEGF) injection has been shown to improve the success rates of trabeculectomy by decreasing inflammation and neovascular activity.

Ocular surface disease in glaucoma may either be a pre-existing condition or may be exacerbated by the use of multiple topical medications, especially in refractory glaucoma. The prevalence of ocular surface disease in patients with glaucoma has been reported to be up to 50-60%, which may be significantly underdiagnosed [4]. The prevalence and severity of ocular surface diseases tend to increase with the duration of glaucoma treatment and the number of medications used [3].

The following is the pathophysiology of persistent epithelial defect and corneal dysfunction in glaucoma, including NVG.

Drug-induced ocular surface toxicity: Prolonged use of glaucoma medications is commonly associated with ocular surface dysfunction and, in some cases, surface toxicity. These drugs may damage the lacrimal functional unit through a myriad of mechanisms, which include squamous metaplasia, decreased goblet cell density of the conjunctiva, meibomian gland dysfunction and atrophy, increased production of proinflammatory cytokines (e.g., IL-1,6,8, TNF alpha) causing drug-induced cicatrizing conjunctivitis (DICC), and an imbalance of matrix metalloproteinases and tissue inhibitors of metalloproteinases, which lead to tear film instability [10,11]. Glaucoma medications that contain preservatives can further damage the ocular surface in these NVG patients, especially since most will require long-term aggressive treatment for IOP control. In addition to these mechanisms, an allergic reaction to certain glaucoma medications or their preservatives may also contribute to ocular surface damage.

Glaucoma-associated endothelial dysfunction: Decreased endothelial cell density has been reported across studies in glaucoma patients - with patients with higher IOPs known to be more susceptible to endothelial damage. This may be due to direct mechanical stress caused by high IOP, leading to endothelial cell loss and dysfunction [12]. Once the endothelial cell count declines below a critical threshold, corneal dehydration cannot be maintained, resulting in edema and loss of transparency. The associated edema may further impair epithelial adhesion, particularly in diabetic patients, thereby predisposing them to epithelial defects. Additionally, glaucoma medications and antifibrotic agents such as MMC and 5-fluorouracil (5FU) may cause endothelial dysfunction.

Neurotrophic keratopathy: Corneal innervation impairment may lead to decreased corneal sensitivity, which may further lead to punctate keratopathy, epithelial erosions, and persistent epithelial defects. This innervation impairment may occur in NVG patients due to chronic topical medication use, which includes chronic preservative (specifically Benzalkonium chloride) exposure, or may be a result of direct damage due to the raised IOP [13]. In these patients, corneal sensitivity may further decline following cataract or glaucoma surgeries, as well as in the presence of systemic conditions, such as diabetes, which are commonly present.

Anterior segment ischemia in NVG: Common causes for NVG include diabetic retinopathy, central retinal vein occlusion, and ocular ischemic syndrome. The primary pathology in NVG is retinal hypoxia, which disrupts the balance between proangiogenic and anti-angiogenic factors. VEGF, produced by retinal cells and the non-pigmented ciliary epithelium, is the principal proangiogenic factor implicated in NVG, driving neovascularization of the iris and angle [8,14]. It is postulated that oxygen from the aqueous diffuses posteriorly towards the ischemic retina, thereby causing iris and anterior segment ischaemia [8]. This results in further production of VEGF by the ciliary epithelium, along with disrupting the epithelial and endothelial cell integrity. This may be an additional factor responsible for ocular surface dysfunction.

Limbal stem cell deficiency: Following an insult causing epithelial defect, the limbal stem cells and epithelial cell migration are crucial for the regeneration of tissue. Therefore, factors that cause limbal stem cells deficiency may cause persistent epithelial defects and neovascularisation [15]. These commonly include causes such as alkali injuries but may also occur iatrogenically following limbal filtering surgeries using MMC and 5FU [16].

Diabetes mellitus: The cornea is densely innervated by the ophthalmic branch of the trigeminal nerve. Systemic conditions such as diabetes can damage these corneal nerves, resulting in epithelial dysfunction, reduced blink rate, and diminished tear production, ultimately leading to persistent epithelial defects [15].

Mechanical: Most glaucoma patients include the elderly population, which may have tremors, and improper installation technique of the drug is a known problem in these patients [17]. These drug administration techniques can cause repeated microtrauma to the cornea, requiring continual epithelial regeneration. This ongoing injury may serve as a significant risk factor for the development of persistent epithelial defects in these patients [15]. Table 1 presents the mechanisms of persistent epithelial defects in neovascular glaucoma.

**Table 1:** Mechanisms of persistent epithelial defects in neovascular glaucoma. IL: interleukin, TNF: tumor necrosis factor, DICC: drug-induced cicatrizing conjunctivitis

Etiology	Mechanisms
Drug-induced surface toxicity	Squamous metaplasia, decreased goblet cell density, Meibomian gland dysfunction & dropouts, increased proinflammatory cytokines (e.g., IL-1,6,8, TNF alpha), imbalance of matrix metalloproteinases & tissue inhibitors of metalloproteinases, DICC, tear film instability
Glaucoma-associated endothelial dysfunction	Higher IOP associated with increased endothelial loss (due to mechanical stress), leading to epithelial edema and epithelial defects
Neurotrophic keratopathy	Loss of corneal innervation - Local or systemic damage (Diabetes) to the trigeminal nerve
Anterior segment ischemia	Retinal hypoxia stimulates VEGF production, diffusion of oxygen from aqueous towards retina, resulting in anterior segment ischemia and subsequent loss of epithelial and endothelial cell integrity
Limbal stem cell deficiency	Hamper epithelial regeneration
Diabetes	Damage to corneal nerves, causing epithelial dysfunction, reduced blink rate
Mechanical	Recurrent abrasions (following mechanical trauma) can cause depletion of epithelial stem cells

The possible cause of the persistent epithelial defect, which resolved following surgical management, in this case, may be drug-induced corneal toxicity due to long-term use of glaucoma medications. Ocular antihypertensive medications are known to cause adverse effects such as decreased basal tear production, meibomian gland disease, and chronic inflammatory response, along with dry eye disease, which may further be exacerbated by the preservatives commonly used in their formulations [3,7,10]. All these factors, along with the diabetic status of the patient, may have predisposed to an epithelial defect that did not resolve despite a bandage contact lens application. The presence of a persistent epithelial defect in both eyes with uncontrolled IOP on maximum medical therapy made this case more challenging.

The management of persistent epithelial defects typically follows a stepwise approach, beginning with conservative measures such as frequent instillation of preservative-free lubricating eye drops (administered hourly or every two hours) and the application of soft bandage contact lenses. In cases where initial therapy is insufficient, temporary tarsorrhaphy may be employed to facilitate epithelial healing [15]. In this case, initially patient was shifted to preservative-free glaucoma medications, frequent lubricating eye drops, along with BCL application; however, the defect failed to heal. In cases refractory to conservative measures, like this one, surgical measures such as amniotic membrane grafting with debridement need to be considered [15].

The use of amniotic membrane grafting has been established in treating persistent epithelial defects [15,18]. The amniotic membrane graft serves as a scaffold for re-epithelialization while providing growth factors that promote healing along with anti-inflammatory effects. The use of preservative-free medications, particularly tear substitutes, in the post-operative period can further help maintain hydration and provide factors essential for epithelial recovery in these cases. Additionally, amniotic membrane grafting has been used to improve the success rates in trabeculectomies under the scleral flap placement in refractory glaucoma cases [19]. However, its use in this case of neovascular glaucoma with persistent epithelial defect is unique, highlighting the importance of a comprehensive approach while managing such complex cases.

## Conclusions

This case report describes a patient who presented with an intractable form of neovascular glaucoma, which could not be medically managed. This was further complicated by the presence of a persistent epithelial defect, which occurred as a result of the compromised ocular surface following long-term use of multiple glaucoma medications and the diabetic status of the patient.

The presence of uncontrolled neovascular glaucoma, presenting with a persistent epithelial defect, presented a unique management challenge. Amniotic membrane grafting along with trabeculectomy helped heal the poor ocular surface along with achieving an adequate IOP control. A multimodal approach of co-managing neovascular glaucoma and the corneal pathology resulted in favorable outcomes while salvaging vision in both eyes. This case highlights the importance of a tailored approach - comanaging corneal and glaucomatous pathology.
